# Evolutionary analysis and interaction prediction for protein-protein interaction network in geometric space

**DOI:** 10.1371/journal.pone.0183495

**Published:** 2017-09-08

**Authors:** Lei Huang, Li Liao, Cathy H. Wu

**Affiliations:** 1 Department of Computer and Information Sciences, University of Delaware, Newark, DE, United States of America; 2 Center for Bioinformatics and Computational Biology, University of Delaware, Newark, DE, United States of America; King’s College London, UNITED KINGDOM

## Abstract

Prediction of protein-protein interaction (PPI) remains a central task in systems biology. With more PPIs identified, forming PPI networks, it has become feasible and also imperative to study PPIs at the network level, such as evolutionary analysis of the networks, for better understanding of PPI networks and for more accurate prediction of pairwise PPIs by leveraging the information gained at the network level. In this work we developed a novel method that enables us to incorporate evolutionary information into geometric space to improve PPI prediction, which in turn can be used to select and evaluate various evolutionary models. The method is tested with cross-validation using human PPI network and yeast PPI network data. The results show that the accuracy of PPI prediction measured by ROC score is increased by up to 14.6%, as compared to a baseline without using evolutionary information. The results also indicate that our modified evolutionary model DANEOsf—combining a gene duplication/neofunctionalization model and scale-free model—has a better fitness and prediction efficacy for these two PPI networks. The improved PPI prediction performance may suggest that our DANEOsf evolutionary model can uncover the underlying evolutionary mechanism for these two PPI networks better than other tested models. Consequently, of particular importance is that our method offers an effective way to select evolutionary models that best capture the underlying evolutionary mechanisms, evaluating the fitness of evolutionary models from the perspective of PPI prediction on real PPI networks.

## Introduction

With continuous efforts in identifying protein-protein interactions (PPIs) through both high-throughput wet-lab experiments and computational methods, an increasing number of new PPIs have been discovered and validated, enabling sizeable (even genome wide) PPI networks to be formed. Consequently, researchers have begun to devote more attention to study PPI networks, aiming at better understanding biological function of individual proteins, protein complexes and even larger subcellular machines as a complex system [1].

One important forefront in PPI network study is to understand how the PPI networks evolve over time. In order to reveal the underlying evolutionary mechanism of PPI network, many evolutionary models, such as Duplication-Divergence model [2–5], Scale Free model [6] etc., have been proposed to simulate the evolutionary processes of PPI networks. For these different evolutionary models, there are still some controversies about fitting models to different species [7–9]. The uncertainty of evolutionary models is attributed to several factors, including the incompleteness of the current PPI networks, ineffective evaluation methods and limited applications. While recent efforts have led to development of more effective evaluation methods, such as using an Approximate Bayesian computation and Differential Evolution based method [10], the evalution is essentially restricted to using simulated data, primarily due to the lacking of “ground truth”—the ancestral networks—for real PPI network data. On the other hand, although evolutionary models are mainly used to explain how the networks evolve from an ancient version to what they currently are, by going back in time, namely “removing” edges from the current networks, it would be also useful, probably even more so, to let us “add” edges, i.e., to make prediction of de novo interactions.

On the other hand, some attempts at de novo interaction prediction based on PPI network topology have been made, using techniques that can be loosely categorized as “node neighborhood” [11–13]: assign a likelihood score to any candidate interaction—a pair of non-connected proteins in a PPI network—based on the topological properties of neighboring nodes. It is known that these methods perform poorly when the networks are sparse and noisy [12]. Higham et al. [14] suggest that PPI networks may have a geometric random structure. And most recently, Zhu et al. [15] further support the hypothesis of geometric random structure through recovering the PPI network by geometric embedding. Moreover, geometric embedding based methods [12, 16, 17] are demonstrated to be helpful for de-noising PPI networks. Although the embedding based methods are rooted in the notion that a PPI network lies on a low dimensional manifold shaped in a high-dimensional space whose topology is determined by the constraints imposed on the protein interactions through biological evolution, no evolutionary models are explicitly invoked.

In this work, we developed a novel method that combines evolutionary models and geometric embedding to make prediction of de novo protein interactions in a PPI network. The key idea of our method is to apply an evolutionary model onto a partial PPI network (with some edges reserved for testing) and embed the evolved network into a geometric space, then to predict new interactions based on Euclidean distance in that geometric space. The cross-validation using human PPI network and yeast PPI network data indicates that the invocation of appropriate evolutionary model can increase the accuracy of PPI prediction measured by ROC score by up to 14.6%, as compared to a baseline without using evolutionary information. Our approach also provides means for assessing evolutionary models on real PPI network data. The results show that our modified evolutionary model—combining a gene duplication/neofunctionalization model and scale-free model—has a better fitness and prediction efficacy for these two PPI networks.

## Materials and methods

### Overview

A PPI network can be conveniently represented as a graph *G* = (*V*, *E*) with *V* nodes (proteins) and *E* edges (interactions). G is defined by the adjacency matrix *A* with *V* × *V* dimension:

$$\begin{array}{c} a_{i,j}=\{\begin{array}{c} 1,if\left(i,j\right)\in E\\ 0,if\left(i,j\right)\notin E \end{array} \end{array}$$ (1)

where *i* and *j* are two nodes in the nodes set *V*, and (*i*, *j*) represents an edge between *i* and *j*, (*i*, *j*) ∈ *E*. The graph is called *connected* if there is a path from any nodes to any other nodes in this graph. The real PPI networks are usually incomplete due to limitation of current experimental results and thus are disconnected. But in practice a maximum connected component that includes most of the nodes and edges can be found. For example, the yeast PPI dataset obtained from DIP (Release 20140117) [18] has 5,119 proteins among which 5,055 proteins belong to the maximum connected component. In order to avoid the problem of spatial overlap that is caused by embedding disconnected components, this paper only takes into consideration the maximum connected component.

**Fig 1:**
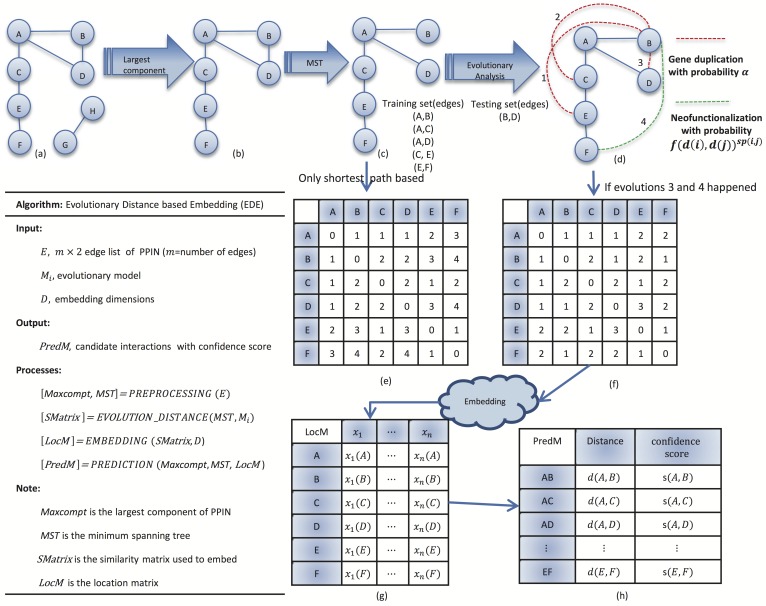
An example to show the principle of our algorithm. (a) Ground-truth network; (b) Maximum connected component; (c) Minimum spanning tree (training set); (d) Evolved network (dash lines are elementary predictions based on evolutionary analysis); (e) Distance matrix based on shortest path in MST; (f) Distance matrix based on evolutionary distance; (g) Coordinates in geometric space; (h) Euclidean distances in geometric space and corresponding confidence scores.

The flow of our algorithm is generally illustrated in Fig 1. Given a PPI network, we first find the maximum connected component and its minimum spanning tree (MST) by Prim algorithm [19]. Note that the MST is not unique in our study because all the edges are assigned equal weight of 1. Then MST is used as the training sub-network. The rest of edges that are not in the MST will be used as a testing set to evaluate the performance of the predictions. Based on the training sub-network, the distances between all pair of nodes can be obtained to form a distance matrix. In typical embedding methods, the distance is the shortest path between node pairs in the minimum spanning tree, which is also called “Minimum Curvilinearity” that proposed by the work of Cannistraci et al. [12, 17]. In this work, we propose a new “distance” called *evolutionary distance*, as we hypothesize that, through evolutionary analysis we can get a more complete training sub-network based on the minimum spanning tree. After applying the evolutionary model, the evolved training sub-network is embeded into a geometric space, where all of nodes are assigned with coordinates and the spatial distance between any node pairs can be computed. Confidence scores are derived from various spatial distances. Lastly, interaction prediction is made for candidate pairs based the confidences scores, and is evaluated by comparing against the testing set.

More formally, given a PPI network with *E* edges, edges in the training sub-network can be represented by set *TrnE* ∈ *MST*, and testing edge set is *TstE* = *E* − *TrnE*, and *TstE* ∩ *TrnE* = ∅. The predicted edges are put into a set called *PE*, and the correct prediction (true positive) is the overlap between sets *PE* and *TstE*: *PE* ∩ *TstE*.

### Network embedding algorithm

Multi Dimensional Scaling (MDS) [20] is a classical nonlinear dimensionality reduction algorithm based on Euclidean distance. While the PPI network is represented by graph without knowing the coordinates of the nodes, so instead of using MDS directly we adopt the extension algorithm isometric feature mapping (Isomap) [21] that based on geodesic distance. However, without considering the difference between distance matrices, MDS and Isomap are equivalent to each other. Therefore, we choose MDS(Isomap) embedding technique for our method. Given a set of *n* nodes and a distance matrix whose elements are shortest paths between all node pairs, the basic idea of Isomap is to find coordinates in a geometric space for all the nodes such that the distance matrix derived from these coordinates approximates the original geodesic distance matrix as well as possible.

More specifically, it essentially takes the following steps to accomplish the embedding task.

1. Construct PPI interaction network(graph), and get the largest connected component.
2. Compute the shortest paths of all node pairs to get matrix **D**.
3. Apply the double centering to **D** and get the symmetric, positive semi-define matrix: $A=-\frac{1}{2}JD^{2}J$, **J** = **I** − *n*^−1^**11**′, where **I** is the identity matrix that has the same size as **D**; and **1** is a column vector with all one, and **1**′ is the transpose of **1**.
4. Extract the *m* largest eigenvalues λ_1_…λ_*m*_ of **A** and the corresponding *m* eigenvectors *e*_1_ … *e*_*m*_, where *m* also means the dimensions of target geometric space.
5. Then, a *m*-dimensional spatial configuration of the *n* nodes is derived from the coordinate matrix $X=E_{m}\Lambda_{m}^{1/2}$, where **E**_*m*_ is the matrix with *m* eigenvectors and Λ_*m*_ is the diagonal matrix with *m* eigenvalues of **A**.

Besides, there are many other embedding algorithms, such as Stochastic Neighbourhood Embedding (SNE) [22] and tSNE [23], Minimum Curvilinearity Embedding (MCE), non-centered MCE (ncMCE) proposed by Cannistraci et al. [12, 17] and so on. It is really hard to tell which one is better than others. Given the primary goal of our paper is to compare the prediction efficacy of different evolutionary distance matrices and further analyze the fitnesses of different evolutionary models respect to the real PPI networks, we only include most recent MCE [17], ncMCE [12] and method proposed by Kuchaiev et al. [16] in our result comparison.

### Evolutionary distance based on different models

Instead of setting up the distance matrix based on shortest path between node pairs in the minimum spanning tree [12, 17], we propose a new evolutionary distance through analyzing the underlying evolutionary relations between node pairs. In practice, various evolutionary models may be needed for this analysis. One common mechanism by which PPI networks evolve is gene duplication; subsequently, another mechanism named post-duplication divergence may cause the PPI network to further evolve.

Briefly, the whole evolutionary process can be described as follows: given an ancestral PPIs network, any node (target node) may be duplicated at a certain probability; and then during the divergence process, some new connections may be formed between the duplication node and the target node and target node’s neighbors. As the time goes on, the PPI networks evolves to its present form. However, in our application, the time arrow is reversed. That is, given a minimum spanning tree of a current PPI network, it consists of all proteins already; no new proteins would be added any more. Instead, we can infer the evolutionary relations between the existing protein pairs, for example, a protein is a duplication of its neighboring protein in the PPI network. Inspired by the most recent evolutionary model DUNE (Gene Duplication and Neofunctionalization model) [5], for any two nodes whose shortest path is 1 or 2 in the minimum spanning tree, they may be duplication for each other. In this work, we analyze the evolutionary relations and compute the evolutionary distance between node pairs based on modified DUNE, named as DANEOsf in Eq (2), where *DANEOsf*_*EvoDist*(*i*, *j*) represents the evolutionary distance between nodes (proteins) *i* and *j*, *SP*(*i*, *j*) represents the shortest path between nodes *i* and *j* in the minimum spanning tree graph, *deg*(*i*) and *deg*(*j*) are the degrees for nodes *i* and *j* respectively, Δ(*MST*) indicates the maximum degree of the minimum spanning tree, *p*_*dup*_ indicates the probability of interaction caused by gene duplication and divergence (*α* = 0.3 in our experiments), and *p*_*neo*_ is the probability of neofunctionalization. Different with the original neofunctionalization proposed by DUNE [5], we introduce the scale-free idea into our neofunctionalization part such that, the larger the degree of a node is, the higher the probability at which the node will initiate neofunctionlization.

Fig 1 shows the whole process of our method, where panels (c) and (d) are for the evolutionary analysis. Given a minimum spanning tree shown by Fig 1(c), take node *B* for example, it can be regarded as the duplication of target nodes {*A*, *C*, *D*}, so there are possible connections between the duplication node *B* and nodes {*A*, *C*, *D*, *E*} caused by gene duplication and divergence (red dash lines result from our evolutionary analysis). Meanwhile, another connection, as shown by green dash line, may be caused by neofunctionalization. After analyzing the evoluationary relations between all node pairs, we can get the evolutionary distance matrix as Fig 1(f) shows. In other words, for any node pairs whose shortest path in MST is larger than 1, there is a predicted interaction between them if their shortest path in the evolved network becomes equal to 1.

As mentioned earlier, since it is not known a priori which evolutionary model best captures the underlying evolutionary mechanisms that a given PPI network has gone through, multiple models will be deployed, and the best one is chosen as the model that delivers the most accurate PPI prediction. In this work, we include the following models for the evolutionary analysis: Linear Preference Attachment (LPA) [6] model Eq (3) and Random Mutation model (RM) Eq (4), where *β* = 0.15 in our experiments. The values of *α* and *β* are set by grid search with optimizing prediction performance on a small validation set.

$$DANEOsf\_EvoDist\left(i,j\right)=\{\begin{array}{ll} if\;SP\left(i,j\right)=1: & 1\\ if\;SP\left(i,j\right)=2,3: & \{\begin{array}{l} 1,\;at\;p_{dup}=\alpha \\ SP\left(i,j\right),\;at\;1-p_{dup} \end{array}\\ if\;SP\left(i,j\right)>3: & \{\begin{array}{l} 1,\;at\;p_{neo}={\left(\frac{deg\left(i\right)deg\left(j\right)}{\Delta {\left(MST\right)}^{2}}\right)}^{SP\left(i,j\right)}\\ SP\left(i,j\right),\;at\;1-p_{neo} \end{array} \end{array}$$ (2)

$$LPA\_EvoDist\left(i,j\right)=\{\begin{array}{ll} if\;SP\left(i,j\right)=1: & 1\\ if\;SP\left(i,j\right)>1: & \{\begin{array}{l} 1,\;at\;p_{lpa}=\frac{1}{2}\frac{deg\left(i\right)deg\left(j\right)}{\Delta {\left(MST\right)}^{2}}\\ SP\left(i,j\right),\;at\;1-p_{lpa} \end{array} \end{array}$$ (3)

$$RM\_EvoDist\left(i,j\right)=\{\begin{array}{ll} if\;SP\left(i,j\right)=1: & 1\\ if\;SP\left(i,j\right)>1: & \{\begin{array}{l} 1,\;at\;p_{rm}=\beta \\ SP\left(i,j\right),\;at\;1-p_{rm} \end{array} \end{array}$$ (4)

### PPI prediction

Each protein in the PPI network is assigned spatial coordinates from the embedding of the network (its distance matrix) into a geometric space by Isomap. Euclidean distance between all protein pairs can then be computed based on the coordinates. A threshold *ϵ* on the pairwise distance can be chosen empirically to determine whether there is an interaction between protein pairs. Namely, if the Euclidean distance between any two points is less than or equal to *ϵ*, there is a predicted interaction between their corresponding proteins in PPI networks. Otherwise, there is no interaction between them.

However, considering the large and sparse PPI network and some noises that might have inevitably “survived” despite the de-noising effect of the embedding process, we assign a confidence score to each prediction as [16] did, instead of strictly predicting node pairs into edges (interactions) and non-edges (non-interactions) only based on their Euclidean distance. As previously described, we divide edges (interactions) of a given PPI network into the training set (edges in MST) and testing set (edges not in MST). The following two probability density functions: *p*(*dist*|*edge*) and *p*(*dist*|*nonedge*) can be learned based on the PPI data. We use an Expectation Maximization (EM) algorithm [24] to construct these two density functions that containing maximum likelihood estimates of the parameters in a Gaussian mixture model.

$$\begin{array}{c} p\left(dist|edge\right)=\sum_{i=1}^{k}\pi_{e,i}EG\left(dist,\mu_{e,i},\sigma_{e,i}^{2}\right) \end{array}$$ (5)

$$\begin{array}{c} p\left(dist|nonedge\right)=\sum_{i=1}^{k}\pi_{ne,i}NEG\left(dist,\mu_{ne,i},\sigma_{ne,i}^{2}\right) \end{array}$$ (6)

The density distributions *p*(*dist*|*edge*) and *p*(*dist*|*nonedge*) are computed by Eqs (5) and (6) respectively. Eqs (5) and (6) are linear combinations of *k* Gaussian distributions, where *k* is the number of Gaussian components in the linear mixture model, *π*_*e*,*i*_, *μ*_*e*,*i*_ and $\sigma_{e,i}^{2}$ are weight, mean and variance for edge Gaussian component *i*; *π*_*ne*,*i*_, *μ*_*ne*,*i*_ and $\sigma_{ne,i}^{2}$ are for nonedge Gaussian component *i*. In our experiments *k* was selected to be 3, since we observed there were at most 3 modes for the histograms corresponding to the densities of *p*(*dist*|*edge*) and *p*(*dist*|*nonedge*). Using Bayes’ rule we can compute the posterior probability of edge and nonedge given a distance. The computing methods for edge and nonedge are shown by Eqs (7) and (8), where *p*(*edge*) and *p*(*nonedge*) represent the fraction estimation of edges and nonedges in the network.

$$\begin{array}{c} p\left(edge|dist\right)=\frac{p(dist|edge)p(edge)}{p(dist)} \end{array}$$ (7)

$$\begin{array}{c} p\left(nonedge|dist\right)=\frac{p(dist|nonedge)p(nonedge)}{p(dist)} \end{array}$$ (8)

Finally, a confidence score of predicted interaction between nodes *i* and *j* can be computed using Eq (9).

$$\begin{array}{c} S_{i,j}=\frac{p(edge_{i,j}|dist_{i,j})}{p\left(edge_{i,j}|dist_{i,j}\right)+p\left(nonedge_{i,j}|dist_{i,j}\right)} \end{array}$$ (9)

Based on the confidence score, we can evaluate prediction performance using receiver operating characteristic (ROC) curve and ROC score, and compare the prediction efficacies among different evolutionary models. For any node pair (i, j) that do not have an edge in the MST, we compute its confidence score *S*_*i*,*j*_ and rank all these node pairs by their confidence score in decreasing order. Moving down this ranked list, set a threshold at any point, then node pairs above that threshold are called positive, i.e., edges are predicted for these node pairs. These predicted edges are considered true posive (TP) if they are indeed present in the testing set, otherwise are considered false positive (FP). ROC curve plots the true positive rate (as Y-axis) against the false positive rate (as X-axis) as the threshold moves from the top to the bottom of the ranked list. This curve allows visualizing the performance of a predictor: at any given false positive rate, how many true positives are predicted, therefore, a higher curve corresponds to a better performance. ROC score is the area under the curve has a range of [0, 1], with 1 being the best performance. In our study, ROC curve and score evaluate how well a method can recover these existing edges in the network that are reserved for testing. The capability of recovering these existing edges as true positive in such a cross-validation setting can therefore be indicative of a method’s predictive power for detecting de novo edges, although truly de novo edges can only be validated experimentally. Caveats of performance evaluation using cross-validation due to incompleteness of and noise in the current networks are discussed in the Discussion subsection.

Note that biological significance evaluations, like the GO-term based evaluation in Cannistraci et al [12], are typically used when there is no direct evidence about the existence of these predicted de novo edges. As a surrogate to the ground truth, biological properties/functions of the two proteins are used to determine confidence/significance of the predicted edge (PPI) between these proteins. For example, in Cannistraci et al [12], the GO-based evaluation uses semantic similarity between the GO-terms associated with the two proteins to decide how biologically relevant or likely to have an interaction between these two proteins, and hence to “quantify the precision of the predictors.” Note that, however, the reliability of such GO-term semantic similarity is itself evaluated by positive and negative protein interaction sets from databases such as DIP, and remains an active research problem [25].

### Data

We use yeast and human PPI networks downloaded from DIP (Release 20140117. http://dip.doe-mbi.ucla.edu/dip/Download.cgi) [18] and HPRD (Release 9. http://hprd.org/download) [26] to test our algorithm. The detail information of these two datasets is shown in Table 1. Obviously, PPI networks of yeast and human are very large and sparse, especially serious for human PPI network that includes 9,673 nodes while only 39,240 known interactions. The maximum connected component of human PPI network only has 9,270 proteins, which is less than half of total. For yeast PPI network, the maximum connected component includes most of the network’s nodes and edges. For these two data sets, PPI prediction here means that we need to find out the *TstE* (true interactions) from all the candidate interactions (*CdtE*). Given the ratios of *TstE* to *CdtE* for yeast and human data sets are very small, it makes the prediction task very difficult, especially for human.

**Table 1:** PPI network information.

Species	Nodes	Edges	Nodes(MaxComp)	Edges(MaxComp)	TrnE	TstE	CdtE
Yeast	5,119	22,637	5,055	22,285	5,054	17,231	12,768,931
Human	9,673	39,240	9,270	18,459	9,269	9,190	42,952,546

## Results

### Embedding dimension

**Fig 2:**
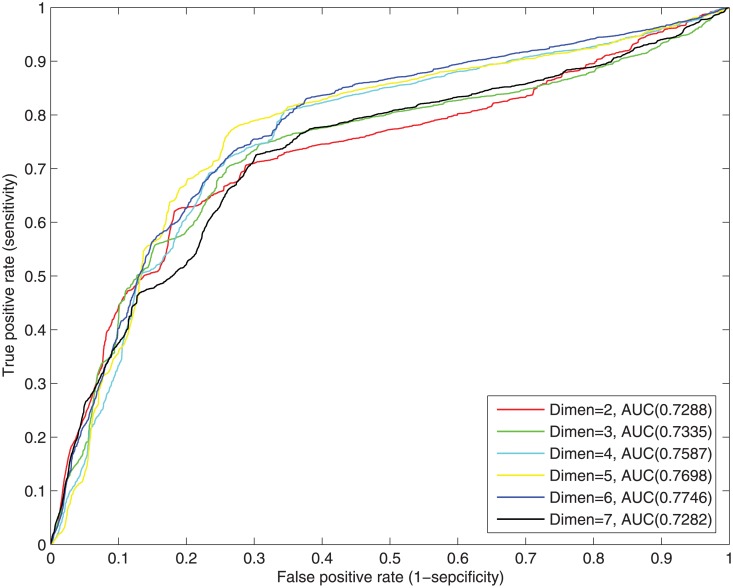
ROC curves of different embedding dimension.

We carry out experiments to demonstrate whether the independent variable, i.e., the dimension of the geometric space, would affect predicting efficacy. We embed the evolved training sub-network of the yeast PPI network into an *n*-dimensional (*n* = 2, 3, 4, 5, 6, 7) geometric space, then use the testing edge set *TstE* to evalulate the prediction efficacy. As shown in Fig 2, dimension can affect the performance, though somewhat slightly: the ROC score grows slowly, from 0.7288 to 0.7746, as dimension increases, and begin to decrease when dimension reaches 7. Therefore, in order to strike a balance between computational cost and prediction efficacy, dimension 5 was chosen to do the following experiments.

### Evolutionary analysis and interaction prediction for real PPI networks

As mentioned in the method section, our method consists of two prediction stages: elementary prediction based on evolutionary analysis and final prediction based on Euclidean distance. To make a comprehensive comparison, we apply our evolutionary distance based embedding (EDE) algorithm to the three evolutionary distance matrices (DANEOsf, LPA and RM) to compare their prediction efficacy; Moreover, we use the minimum spanning tree based shortest path matrix (SP) as an input to the minimum curvilinear embedding algorithms MCE-MDS, MCE-SVD and ncMCE-SVD (Multidimensional Scaling, Singular Value Decomposition and None-centered Singular Value Decomposition versions) proposed by Cannistraci et al. [12, 17], and the embedding method proposed by Kuchaiev et al. [16]. As mentioned in [12], the basic idea of the embedding method adopted by Kuchaiev et al. [16] is equivalent to Isomap [21]. In other word, it is quite similar to MCE-MDS if we use SP as the input distance matrix. However, given the software of Kuchaiev et al. [16] is independently implemented, we include it in our comparison by using the software directly. As shown in Table 1, our experiments use 17,231 interactions as testing set and only 5,054 interactions as the training set for yeast, and 9,190 interactions as the testing set and only 9,269 as the training set for human PPI network.

**Fig 3:**
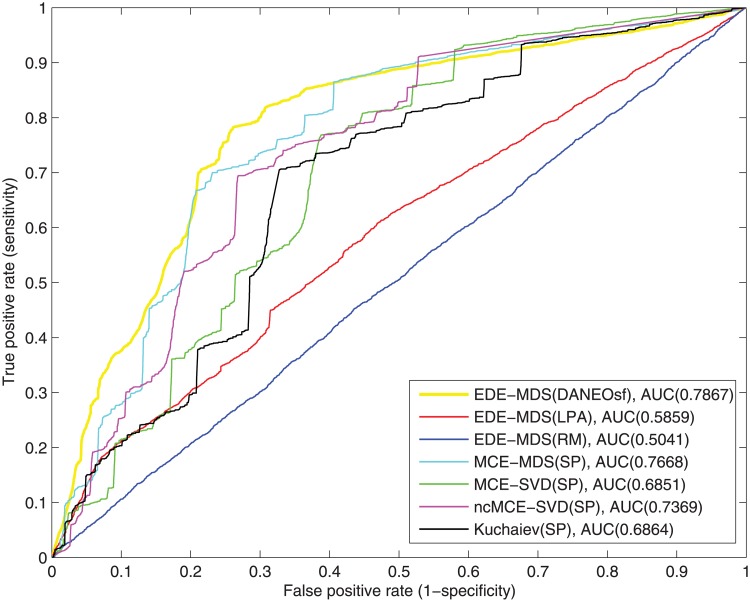
ROC curves of interaction prediction for yeast.

To clearly show the results of different methods and input distance matrices, as shown in the Table 2 and Figs 3∼8, we name each result in the format of AAA-BBB(CCC), where AAA indicates the method name, BBB indicates the embedding techniques without considering the difference of input matrices and CCC indicates the input distance matrix. For the results of yeast PPI prediction, as illustrated in Fig 3, the DANEOsf based prediction EDE-MDS(DANEOsf) has the best ROC curve and highest ROC score that reaches 0.7867. The ROC score of SP based prediction for MCE-MDS, MCE-SVD ncMCE-SVD and Kuchaiev are 0.7668, 0.6851, 0.7369 and 0.6617 respectively. However, EDE-MDS(LPA) and EDE-MDS(RM) are significantly less or almost no predictive power; especially for EDE-MDS(RM), it only has a ROC score of 0.5041, and EDE-MDS(LPA)’s ROC score is 0.5859. This means that poor evolutionary models bear no useful information but a lot of noise, and consequently lead to get poor predictions. Moreover, compare with the traditional method (Kuchaiev) [16] whose ROC score is 0.6864, our DANEOsf based prediction gets a remarkable improvement with 0.1003 (14.6%) increase on ROC score.

**Fig 4:**
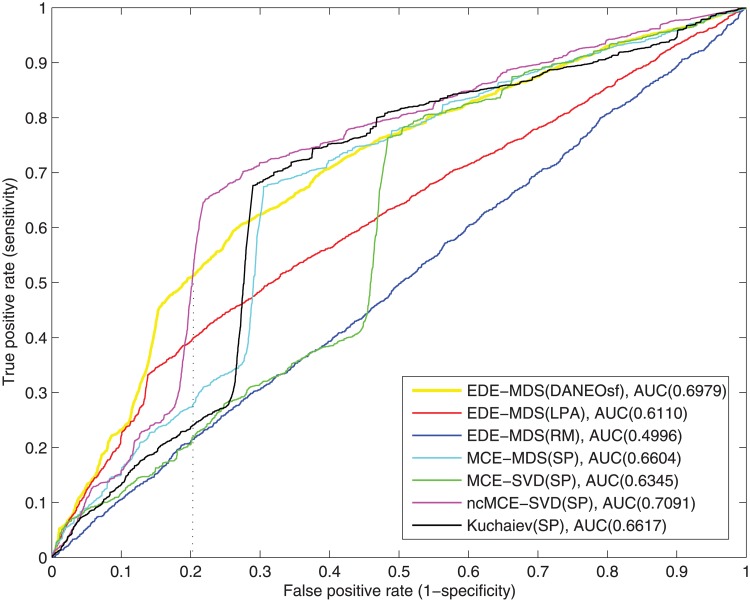
ROC curves of interaction prediction for human.

**Fig 5:**
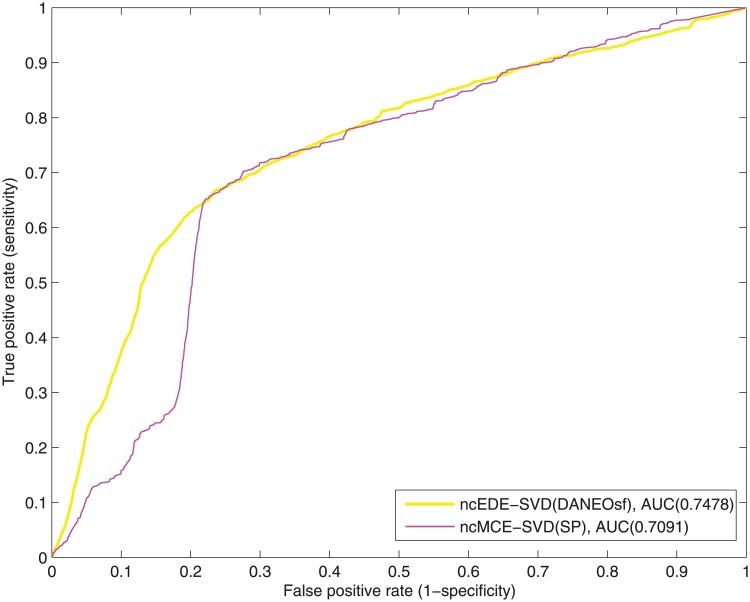
Extra comparison between ncECE-SVD(DANEOsf) and ncMCE-SVD(SP) on interaction prediction for human.

**Fig 6:**
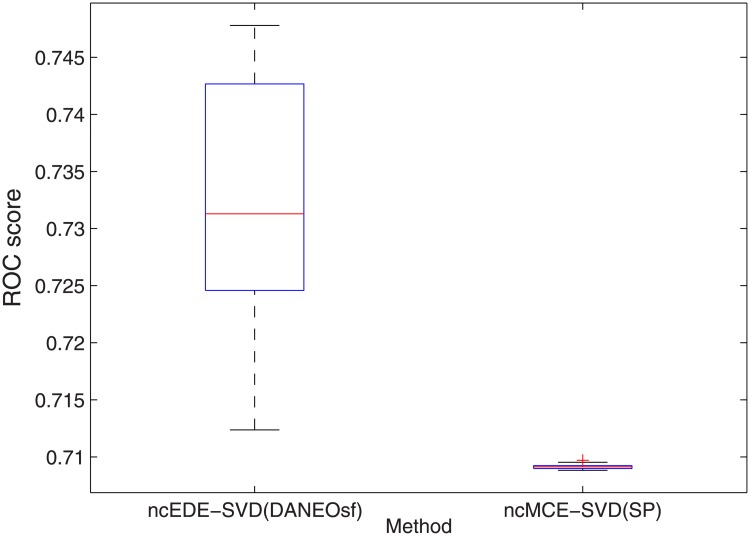
Box plot of ROC scores for human PPI prediction based on ncECE-SVD(DANEOsf) and ncMCE-SVD(SP).

**Fig 7:**
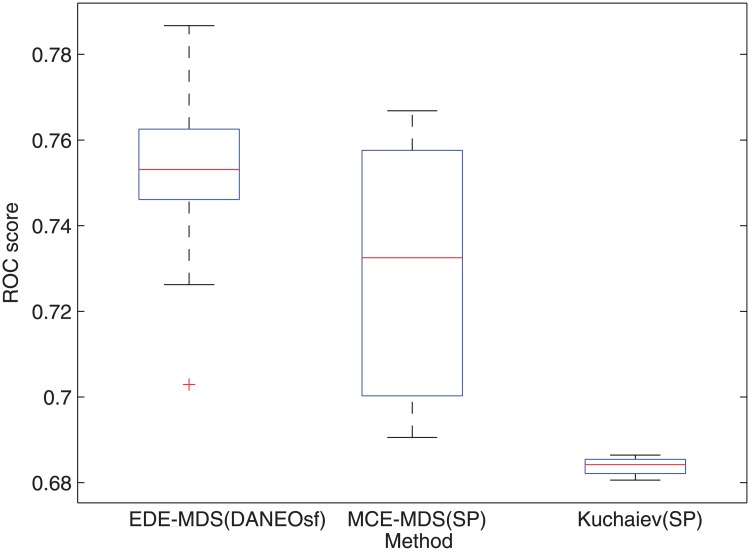
Box plot of ROC scores for yeast PPI prediction based on EDE-MDS(DANEOsf), MCE-MDS(SP) [12] and Kuchaiev [16].

**Fig 8:**
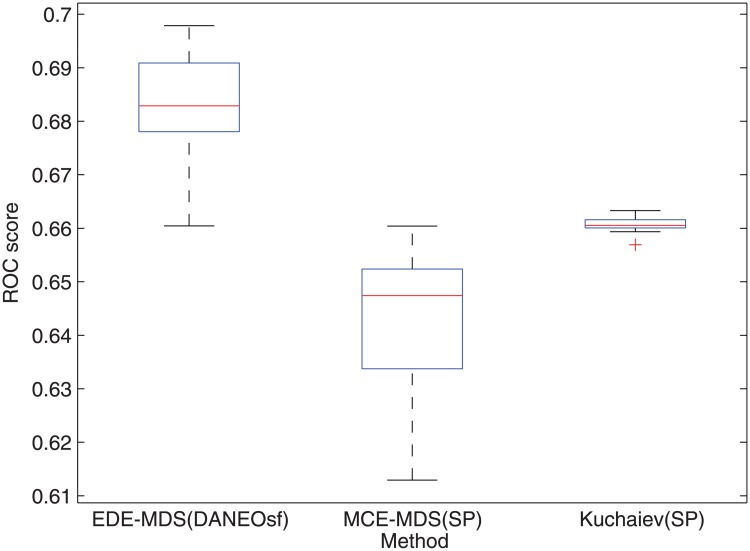
Box plot of ROC scores for human PPI prediction based on EDE-MDS(DANEOsf), MCE-MDS(SP) [12] and Kuchaiev [16].

**Table 2:** P values of paired-sample t-Test for ROC score vectors.

P Values	*Yeast*		*Human*	
	$\underset{\left(\bar{AUC}=0.7308\right)}{MCE-MDS(SP)}$	$\underset{\left(\bar{AUC}=0.6857\right)}{Kuchaiev(SP)}$	$\underset{\left(\bar{AUC}=0.6430\right)}{MCE-MDS(SP)}$	$\underset{\left(\bar{AUC}=0.6609\right)}{Kuchaiev(SP)}$
*Yeast* $\underset{\left(\bar{AUC}=0.7520\right)}{EDE-MDS(DANEOsf)}$	0.0237	5.1684e-09	−	−
*Human* $\underset{\left(\bar{AUC}=0.6835\right)}{EDE-MDS(DANEOsf)}$	−	−	5.2605e-10	7.8320e-07

Fig 4 shows the ROC curves for the human PPI network. Because of its significantly larger size and sparsity, the performance for the human PPI network is generally not as good as that of yeast. Still, it can be seen clearly that our DANEOsf based prediction EDE-MDS(DANEOsf) gets high ROC score of 0.6979. Especially, when false positive rate *FPR* ≤ 0.2 as shown by the dash line in Fig 4, our EDE-MDS(DANEOsf) shows a clear advantage over other models.

However, in Fig 4, the ROC score of ncMCE-SVD(SP) is even higher than that of our EDE-MDS(DANEOsf). Given the ROC socre of ncMCE-SVD(SP) is obvious lower than that of our EDE-MDS(DANEOsf) in Fig 3 for yeast PPI network, we believe the performance of various embedding techniques may be sensitive to the difference PPI networks. So we make an extra comparison by using non-centered SVD as the embedding technique for our DANEOsf evolutionary distance matrix, we name the result as ncEDE-SVD(DANEOsf). The comparison between ncEDE-SVD(DANEOsf) and ncMCE-SVD(SP) is shown in Fig 5, which shows the ncEDE-SVD(DANEOsf) has a higher ROC score and gets an improvement with 5.4% increase on ROC score; and the box plot in Fig 6 based on 15 repeated experiments further demonstrates the stable advantage of ncEDE-SVD(DANEOsf).

Overall, we have demonstrated that our method provides a useful tool at the network level to make de novo PPI prediction for both yeast and human PPI networks.

To make more detailed comparisons, 15 repeated experiments of PPI prediction, involving all the steps described in the algorithm EDE in Fig 1, have been conducted for each methods; due to the equal weight assigned to edges, each experiment may get a different minimum spanning tree as the training network. In the 15 repetitions, only MCE-MDS(DANEOsf), MCE-MDS(SP) and Kuchaiev(SP) are included. For one reason, it has been demonstrated that the performance of different embedding algorithms are sensitive to different PPI networks. A fair comparison among various distance matrices should be based on same embedding techniques (MDS). For another, as shown in Figs 3 and 4, the three ROC scores respectively obtained by EDE-MDS(DANEOsf), MCE-MDS(SP) and Kuchaiev(SP) are quite close to each other. Figs 7 and 8 are the box plots of ROC scores of EDE-MDS(DANEOsf), MCE-MDS(SP) and Kuchaiev(SP) for yeast and human PPI prediction. It clearly shows that the EDE-MDS(DANEOsf) performs stably and better than MCE-MDS(SP) and Kuchaiev(SP). Moreover, we conducted paired-sample t-Test by making null hypothesis that the pairwise difference between ROC score vectors (EDE-MDS(DANEOsf), MCE-MDS(SP) and Kuchaiev(SP)) has a mean equal to zero, the result of t-Test reject the null hypothesis at 5% significance level. And the P values are shown by Table 2.

**Fig 9:**
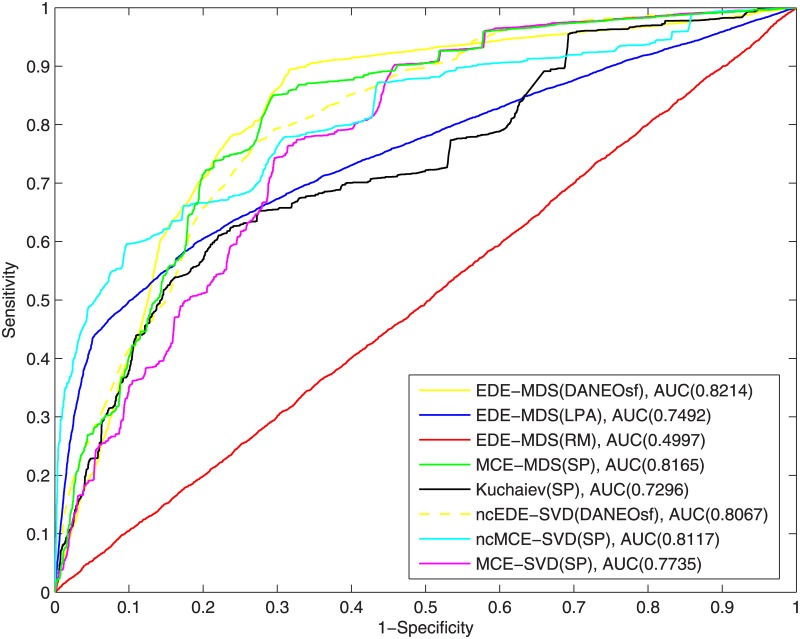
ROC curves of whole network recovery for yeast.

**Fig 10:**
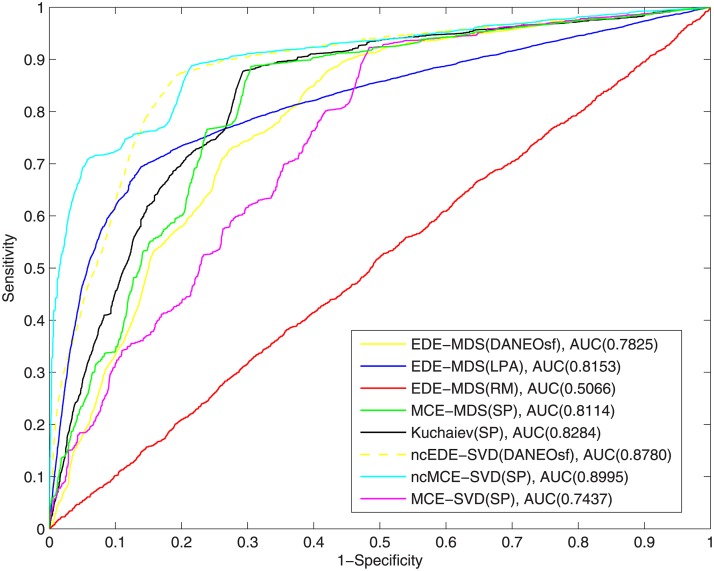
ROC curves of whole network recovery for human.

In the literature for network recovery work, notably Cannistraci et al [12] and Hulovatty et al [27], the evaluation is carried out somewhat differently. As far as we can tell, the key difference between theirs and ours in calculating ROC is how the positive set is created. In their approaches, all the existing edges in the original network are used as a positive set when evaluating the prediction performance, in other words, the positive set include both training and testing edges. In our approach, following the standard procedure for cross-validation, we split the existing edges into two subsets: edges in a minimum spanning tree are used as training and all other edges are reserved as testing, and evaluation of performance is only carried out on the edges in the testing set that are not seen by the method during training. Nonetheless, in the second round of revision, we also recalculated ROC curves and scores by using the entire network as positive set, and the new results are included in Figs 9 and 10 for yeast and human PPI networks respectively.

Fig 9 for Yeast data shows that our method ranks 1st (with ROC = 0.8214), while Fig 10 for Human data shows our method ranks 2nd (with ROC = 0.8780)—ncMCE−SVD(SP) ranks 1st (with ROC = 0.8995). The results show that, as generally expected, all methods fare better when the positive set includes all existing edges as compared to when the positive test only includes existing edges that are reserved for testing and not seen during the training. However, from a machine learning perspective, what is more important is to test model’s ability to generalize by evaluating its performance on a set of data not used for training. In this sense, the fact that the control method ncMCE−SVD(SP) performs better than our method in human PPI network when training data is included but performs worse than our method when tested only on unseen data is an indication that the method may suffer from over-fitting. In other words, our method demonstrates a better generalization, which is especially important for de novo link prediction than network recovery.

**Fig 11:**
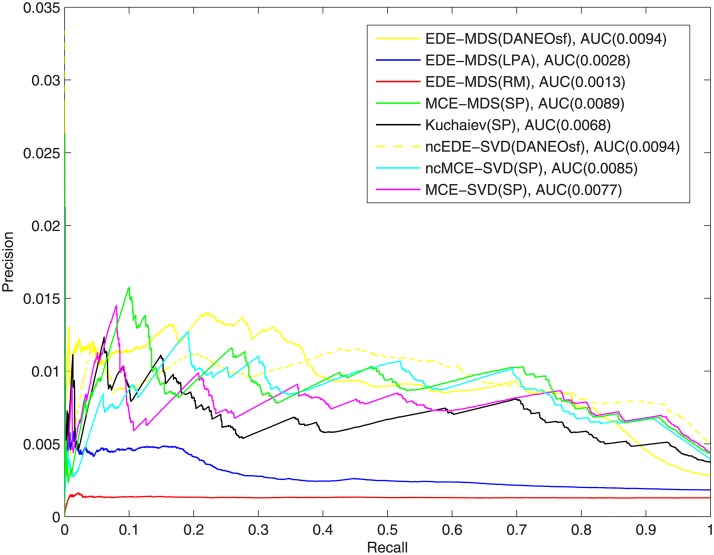
Precision-Recall curves of interaction prediction for yeast.

**Fig 12:**
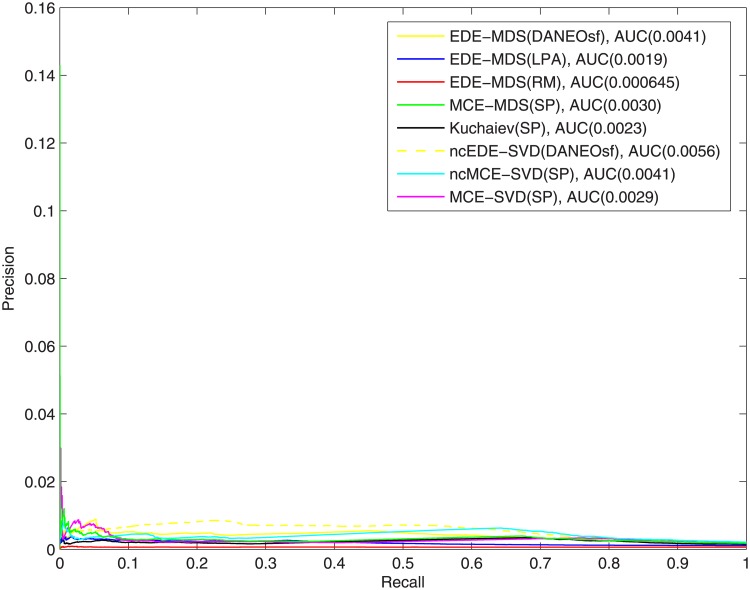
Precision-Recall curves of interaction prediction for human.

Additionally, we plotted precision-recall curves and calculated area under these curves (AUCPR) to gain more insight about the quality of the prediction, shown in Figs 11 and 12. Due to the significant skew of the data—there is far more negatives than positives, admittedly, the absolute values of AUCPR are very low for all methods in this study. Still our method outperforms the control methods in both yeast and human PPI networks, which is consistent with what is shown in ROC evaluation. This comes no surprise as the ROC curves for our method dominate in ROC space, which, as proved in Davis and Goadrich [28], guarantee their dominance in Precision-Recall space as well.

For the causes of the reported low precision, besides the incompleteness and unbalance in the data, it is very plausible that, from the evolutionary perspective, some newly added PPIs might be deleterious and eventually get lost due to the selective pressure, contributing to high false positive. However, it is practically difficult for evolutionary models to simulate the selective pressure and restore the whole evolutionary history. Because of this difficulty, in our method we did not try to simulate the evolutionary process from the scratch, but just instead add more potential interactions to the training network based on evolution mechanisms. So in this case, we effectively assume these interactions that are predicted based on evolution models are the survivors after selective pressure. Although some of them might be false positives, our experiments prove that good evolution models can help improve PPI prediction.

Other possible causes for the low precision may be from the embedding process. The distance matrices Fig 1(e) and 1(f) probably cannot capture the interacting/non-interacting relations good enough for protein pairs. So in the future, it would be worthwhile to investigate and study novel methods (e.g. diffusion distance) to calculate the distance matrices. Also, as shown in our experiments, the prediction performance is sensitive to different embedding techniques. This may indicate that even the distance in high dimensional space cannot differentiate true positive and false positive interactions good enough, since many protein pairs may have very close or even same distances.

## Discussion

Note that, given the incompleteness of the current PPI networks, the evaluation of prediction may be biased, because we do not know which missing links are truly negative and which of them are undetected links or links that will appear in the future. It is likely that the actual prediction performance may be even better than what is shown here, as what is considered as false positives (i.e., predicted edges are not present in the testing set) might turn out to be true positive when the predicted interactions are later validated by new experiments. For example, as we further validated the false positives (threshold for confidence score is 0.8) of HPRD by using BioGRID(version 3.2.110) [29] which is newer, the result shows 5 of false positives appeared in the BioGRID(version 3.2.110) and none of the 5 appeared in HPRD before. Therefore, with all these caveats, it is advisable to interpret a method’s performance relative to other methods rather than by its absolute performance on the evaluation metrics. However, what is uncertain is how such changes will impact on the performance of various methods; depending on the data, it is possible that the current top performer may be outperformed by other methods.

Currently, there is still no consensus yet with respect to topological characteristics of PPI networks; while some reported scale-free [8], others did not [30], and various duplication-divergence based evolutionary models [2–5] have been proposed. It is also worth pointing out that, the primary goal of our work is to utilize network evolutionary analysis and geometric embedding to improve the performance of PPI prediction, which is to, based on a partial network, predict whether there is an edge for a given pair of nodes. This is different from reconstructing a network from scratch by simulation based on some evolutionary models. Therefore, we do not consider our method as network reconstruction per se. Of particular importance is that our method offers an effective way to select evolutionary models that best capture the underlying evolutionary mechanisms, evaluating the fitness of evolutionary models from the perspective of PPI prediction on real PPI networks.

## Conclusion

In this work we developed a novel evolutionary analysis and PPI prediction method that can make de novo PPI prediction using information at the network level. We demonstrated that incorporating evolutionary information into PPI networks can help, in some cases very significantly, improve the prediction performance as compared with using just geometric embedding. In addition, our method offers an effective way to select among multiple candidate evolutionary models the one that best captures the underlying evolutionary mechanisms, as measured by the PPI prediction on real PPI networks instead of simulated networks. The improved PPI prediction performance may suggest that our DANEOsf evolutionary model can uncover the underlying evolutionary mechanism for these two PPI networks better than other tested models. As future work, we will further validate our predicted interactions when new release of PPI data becomes available and will continue to analyze new evolutionary models in terms of their interaction prediction efficacy.
